# Risks and benefits of cannabis as a pain control modality in patients with sickle cell disease

**DOI:** 10.46989/001c.90837

**Published:** 2023-12-18

**Authors:** Jeremy W. Jacobs, Brian D. Adkins, Laura D. Stephens, Jennifer S. Woo, Garrett S. Booth

**Affiliations:** 1 Department of Laboratory Medicine Yale School of Medicine, New Haven, CT, USA; 2 Department of Pathology, Division of Transfusion Medicine and Hemostasis The University of Texas Southwestern Medical Center, Dallas, TX, USA; 3 Department of Pathology University of California, San Diego, La Jolla, CA, USA; 4 Department of Pathology City Of Hope National Medical Center, Irvine, CA, USA; 5 Department of Pathology, Microbiology, & Immunology Vanderbilt University Medical Center, Nashville, TN, USA

**Keywords:** Sickle cell disease, cannabis, pain crisis, opioids, marijuana, discrimination, stigma

Sickle cell disease (SCD), a hematologic disorder defined by the production of a dysfunctional hemoglobin variant (hemoglobin S, HbS), causes vascular occlusions due to sticky, sickled erythrocytes, resulting in severe, recurrent pain crises, and chronic pain.^1^ Individuals with SCD often require chronic analgesia to alleviate debilitating sickle cell-associated pain. However, pain management in this population is a major challenge. This is due to both biological factors, including the multitude of complex, poorly understood pathophysiologic mechanisms underlying the pain, and sociological factors, such as stigma, discrimination, and inherent challenges in navigating the complex, fragmented healthcare system in the United States (US).^2^

Opioids have historically been, and continue to be, the mainstay for managing pain in patients with SCD. Still, these medications are associated with adverse effects, including tolerance and opioid-induced hyperalgesia.^3,4^ Furthermore, recent reports have shown that patients with SCD are experiencing increasing stigmatization when seeking opioids for pain, as well as an increase in inadequate dosing of opioids by prescribers and lack of alternative pain management modalities,^5^ which may be related to the ongoing opioid epidemic in the US. Notably, patients with SCD experience longer emergency department wait times, and their pain may be less controlled than those without SCD.^6–8^

Given these challenges, many patients with SCD turn to cannabis for pain control. Cannabis is becoming more widely legalized in the US, with medicinal marijuana legal in 37 states and the District of Columbia (72.0% of the US population) and recreational marijuana fully legalized in 20 states (45.7% of the US population).^9^ This offers an increasing ability for researchers to study both its potential therapeutic and adverse effects. Furthermore, the legalization of cannabis in the US may usher in a new strategy for treating SCD-associated pain, allowing patients access to alternative pain control modalities. These policy shifts may contribute to an increase in studies assessing the risks and benefits of cannabis in patients with SCD.

Recent reports from various authors have aptly demonstrated the potential for cannabis to ameliorate SCD-associated pain.^3,10–12^ However, before accepting cannabis as an effective option compared to traditional pain control regimens, researchers and healthcare providers must consider all potential risks and benefits of cannabis in patients with SCD, as this disease is characterized by a unique and highly complex biology that remains incompletely understood. For example, Miodownik and colleagues recently showed that prior cannabis use among patients with SCD was associated with a greater likelihood of bone avascular necrosis (AVN) than in non-users, and the former were more likely to have frequent pain crises and high healthcare utilization.^10^ Conversely, the authors found no difference in acute chest syndrome (ACS) rates, stroke, liver disease, pulmonary hypertension, or priapism. Given these findings, the authors asserted that their study “provides reassurance that, while patients with SCD who have more pain-related comorbidities are more likely to use cannabis, users do not show increased rates of other SCD comorbidities.” These findings are important for advancing the discussion surrounding the use of cannabis in patients with SCD.

One aspect that must be addressed when considering how cannabis might affect patients with SCD focuses on how it impacts healthcare utilization. The available evidence on this is conflicting, as some authors have found decreased hospital admissions^13,14^ and/or decreased emergency department (ED) visits,^13^ some have shown no difference when adjusting for potential covariates,^12^ and still others have shown an increase in hospitalizations due to vaso-occlusive crises.^15^ It is vital to consider the route of administration, as inhalation/smoking may increase the risk of acute chest syndrome and, therefore, the hospitalization rate.^14^ These findings illustrate the need for additional prospective and randomized controlled trials to determine the effect of cannabis use on pain crises and other SCD-related adverse events and how these influence healthcare visits in patients with SCD.

Assessing the impact of cannabis on healthcare use, particularly ED visits, is of particular importance, as up to 50% of patients are transfused because of pain crises.^16^ In the absence of other indications, such as respiratory compromise, transfusion for a pain crisis is not recommended. In addition to the general risks inherent to blood transfusion, the procedure predisposes patients with SCD to higher rates of alloimmunization, particularly in the setting of the pro-inflammatory state associated with vaso-occlusive crises.

A second factor to consider is if cannabis itself may alter the biology of SCD due to its immunomodulatory effects.^3,11,17,18^ As cannabis may have anti-inflammatory properties,^3,18,19^ regular users might have a decreased inflammatory response, even during vaso-occlusive crises, compared to non-users, which theoretically could mitigate some of the adverse effects associated with SCD. Furthermore, while SCD-associated pain has historically been thought to be mediated by tissue injury after vascular occlusion, human and animal studies have suggested that a component of this pain may be related to neuropathic processes.^20^ At this time, our understanding of the immunomodulatory effects of cannabis and the biology of SCD and the associated pain are incomplete; thus, additional investigation into how cannabis use may alter these pain processes is paramount to understanding its potential risks and benefits for patients with SCD.

Despite recent calls to broaden the scope of pain management in patients with SCD, practice within the United States continues to be characterized by puritanical ideas relating to the provision of appropriate pharmacologic pain management.^21^ While opioid dependence remains a major health emergency within the country, there likewise remains continued stigmatization regarding cannabis use.^22^ This is a major hurdle regarding access for patients with SCD-related pain. Although smoking cannabis can contribute to respiratory symptoms, alternative administration routes could enable patients to have improved pain tolerance without the burden of opioid dependence. Outdated opinions about ‘reefer madness’ regarding cannabis use are unfounded, and cannabis should be actively investigated as a novel intervention for SCD-related pain.

In summary, we cannot advocate for or against using cannabis as a pain control modality in patients with SCD. Instead, we attempt to highlight the importance of ensuring that all risks and benefits are considered when assessing the potential for cannabis to relieve pain, as recent studies, including those by Miodownik et al., demonstrate how some of the comorbidities associated with SCD are affected (or not affected) by cannabis use. While cannabis may represent a potentially effective pain-control modality, we encourage additional research into the risks and benefits of cannabis use. Although prior research is sparse, the use of recreational marijuana is high and will likely continue to increase with expanded access; as such, it is vitally important to understand the impact of these substances on outcomes in patients with SCD.

## Authors contributions - CRediT

Conceptualization: Jeremy W. Jacobs (Equal), Brian D. Adkins (Equal), Garrett S. Booth (Equal). Formal Analysis: Jeremy W. Jacobs (Equal), Garrett S. Booth (Equal). Investigation: Jeremy W. Jacobs (Equal), Garrett S. Booth (Equal). Writing – original draft: Jeremy W. Jacobs (Equal), Brian D. Adkins (Equal), Garrett S. Booth (Equal). Writing – review & editing: Jeremy W. Jacobs (Equal), Laura D. Stephens (Equal), Jennifer S. Woo (Equal). Supervision: Garrett S. Booth (Lead).

## Data availability

N/A

## Conflict of interest disclosure

All authors report no conflicts of interest related to this research.

## Ethics approval statement

This study was exempt from institutional review board approval as all data are publicly available, and no personally identifiable information was obtained or reported.

## Patient consent statement

No patients were involved in this study.
